# Endoscope-assisted retrosigmoid approach in hemifacial spasm: our experience^☆^

**DOI:** 10.1016/j.bjorl.2018.03.015

**Published:** 2018-05-09

**Authors:** Giampietro Ricci, Arianna Di Stadio, Luca D’Ascanio, Ruggero La Penna, Franco Trabalzini, Antonio della Volpe, Jacques Magnan

**Affiliations:** a University of Perugia, Permanent Anatomical Dissection Laboratory of Otorhinolaryngology, Perugia, Italy; b University of Perugia, Unit of Otorhinolaryngology, Perugia, Italy; c San Camillo Hospital IRCCS, Venice, Italy; d “Carlo Poma” Civil Hospital, Department of Otolaryngology-Head and Neck Surgery, Mantova, Italy; e Meyer Children Hospital, Otolaryngology Unit, Florence, Italy; f “Santobono-Pausilipon” Children's Hospital, Otology and Cochlear Implant Unit, Regional Referral Centre, Naples, Italy

**Keywords:** Hemifacial spasm, Facial nerve, Nerve decompression, Endoscopic surgery, Quality of life, Espasmo hemifacial, Nervo facial, Descompressão do nervo, Cirurgia endoscópica, Qualidade de vida

## Abstract

**Introduction:**

The use of surgical decompression of facial hemispasm due to the loop in the internal auditory canal is not always accepted due to the risk related to the surgical procedure. Currently a new surgical technique allows surgeons to work in safer conditions.

**Objective:**

To report the results with endoscope-assisted retrosigmoid approach for facial nerve microvascular decompression in hemifacial spasm due to neurovascular conflict. The surgical technique is described.

**Methods:**

We carried out a prospective study in a tertiary referral center observing 12 (5 male, 7 female) patients, mean age 57.5 years (range 49–71) affected by hemifacial spasm, that underwent to an endoscope assisted retrosigmoid approach for microvascular decompression. We evaluated intra-operative findings, postoperative HFS resolution and complication rates.

**Results:**

Hemifacial spasm resolution was noticed in 9/12 (75%) cases within 24 h after surgery and in 12/12 (100%) subjects within 45 days. A significant (*p* < 0.001) correlation between preoperative historical duration of hemifacial spasm and postoperative recovery timing was recorded. Only 1 patient had a complication (meningitis), which resolved after intravenous antibiotics with no sequelae. No cases of cerebrospinal fluid leak, facial palsy or hearing impairment were recorded. Hemifacial spasm recurrence was noticed in the only subject where the neurovascular conflict was due to a vein within the internal auditory canal.

**Conclusions:**

The endoscope assisted retrosigmoid approach technique offers an optimal visualization of the neurovascular conflict thorough a minimally invasive approach, thus allowing an accurate decompression of the facial nerve with low complication rates. Due to the less invasive nature, the procedure should be considered in functional surgery of the cerebellar pontine angle as hemifacial spasm treatment, specially when the procedure is performed by an otolaryngologist.

## Resumo

**Introdução:**

O uso de descompressão cirúrgica do espasmo hemifacial devido ao *loop* no canal auditivo interno nem sempre é aceito devido ao risco relacionado ao procedimento cirúrgico. Atualmente, uma nova técnica cirúrgica permite trabalhar em condições seguras.

**Objetivo:**

Relatar os resultados que obtivemos com a abordagem retrosigmóidea assistida por endoscopia para a descompressão microvascular do nervo facial em casos de espasmo hemifacial devido a conflito neurovascular. A técnica cirúrgica é descrita.

**Método:**

Realizamos um estudo prospectivo em um centro de referência terciária observando 12 pacientes (5M, 7F), com média de idade de 57,5 (intervalo 49-71) anos com espasmo hemifacial submetidos a uma abordagem retrosigmóide assistida por endoscopia para descompressão microvascular. Foram avaliados os achados intraoperatórios, a resolução pós-operatória do espasmo hemifacial e as taxas de complicações.

**Resultados:**

A resolução do espasmo hemifacial foi observada em 9/12 (75%) dos casos nas 24 horas após a cirurgia e em 12/12 (100%) dos indivíduos até 45 dias. Uma correlação significativa (*p* < 0,001) entre a duração do histórico pré-operatório de espasmo hemifacial e o tempo de recuperação pós-operatório foi registrado. Apenas um paciente apresentou uma complicação (meningite), que foi resolvida após administração de antibióticos por via intra venosa sem sequelas. Nenhum caso fístula liquórica, paralisia facial ou deficiência auditiva foi registrado. A recorrência do espasmo hemifacial foi observada em único indivíduo em quem o conflito neurovascular foi causado por um vaso no interior do canal auditivo interno.

**Conclusões:**

A técnica da abordagem retrosigmóidea assistida por endoscopia oferece uma ótima visualização do conflito neurovascular através de uma abordagem minimamente invasiva, permite assim uma descompressão precisa do nervo facial com baixas taxas de complicações. Por ser menos invasivo, o procedimento deve ser considerado na cirurgia funcional do ângulo pontocerebelar como tratamento de espasmo hemifacial, especialmente quando o procedimento é feito por um otorrinolaringologista.

## Introduction

Hemifacial spasm (HFS) is the unilateral, involuntary paroxysmal series of tonic or clonic movement of facial muscles. Primary HFS is caused by an arterial or venous vascular compression of the facial nerve.^1^, ^2^, ^3^, ^4^ Vessel compression is supposed to cause nerve demyelization with consequent alteration in signal transmission, that determines a muscle spasm in the territory innervated by the facial nerve.^1^, ^2^, ^3^, ^4^ Two possible theories of HFS pathophysiology have been reported in the literature: the “central” and the “peripheral” hypotheses. According to the former one, the facial nerve injury, due to the neurovascular impingement, would have a regressive action on facial nucleus, thus causing neural hyperexcitability. On the contrary, according to the “peripheral” hypothesis, clinical symptoms would result from ectopic impulse generation and transmission alteration due to facial nerve demyelization.^4^ Even though no definite evidence has been reported on which theory is the accurate one, both mechanisms likely contribute together to the onset of HFS.

The medical treatment for HFS is Botulin Neurotoxin (BoNT) injection. BoNT blocks calcium mediated release of acetylcholine at the synaptic junction and gives HFS transitory relief in the 85% of cases. Limitations of such treatment are the short duration of symptoms’ relief, the high costs, and the risk of secondary non-responsiveness due to the production of neutralizing antibodies. Other limitations are related to pre-existing neurologic diseases contraindicating neurotoxin injections or possible BoNT interactions with other drugs.^5^ In such conditions, Microvascular Decompression (MVD) represents the only option to achieve HFS resolution and an improvement in a patient's quality of life.^6^, ^7^, ^8^, ^9^, ^10^, ^11^, ^12^

Such curative treatment consists of the surgical decompression of the facial nerve and separation of the offending vessel from nerve by the interposition of Teflon sheet. MDV long-term success rate ranges between 83% and 97%.^2^, ^3^, ^8^ Even though MDV is described as a safe procedure, the frequency of surgical complications reported in the literature is not neglegible.^7^, ^8^, ^9^, ^10^, ^11^, ^12^, ^13^, ^14^ In order to maximize the success rate and reduce the frequency of surgical complications, several authors proposed the association between the traditional microscopic approach and the endoscopic one.^15^, ^16^, ^17^, ^18^, ^19^, ^20^, ^21^, ^22^, ^23^ According to this “combined” technique, the procedure is carried out microscopically while the endoscope is used to better visualize the offending vessel before the decompression and to confirm the detachment of the vessel from the nerve at the end of surgery. Despite the improvement of the positive results attained with the “combined” technique, some postoperative complications, especially cerebrospinal fluid (CSF) leak, have been reported.^15^, ^16^, ^17^, ^18^, ^19^, ^20^, ^21^, ^22^, ^23^ In the attempt to further reduce the complication rate and to confirm the reduced morbidity, we have investigated the results obtained by the endoscope-assisted minimally-invasive or keyhole retrosigmoid approach for MDV in HFS. The surgical technique and results are reported.

## Methods

The protocol was authorized by Ethical Committee of the Hospital Silvestrini, even though no number was released according to Italian health legislation, since this was not considered an experimental study. The study was carried out according to the declaration of Helsinki for human rights.

Between December 2012 and December 2014, 12 (5 male/7 female) patients, mean age 57.5 years (range 49–71), affected by HFS underwent to endoscope-assisted minimally invasive retrosigmoid approach for MVD in the Otolaryngology Department of a tertiary referral center.

The diagnosis of HFS was performed on the basis of subjects’ clinical history and radiological imaging. Magnetic Resonance Imaging (MRI) or angio MRI^8^, ^9^, ^10^, ^11^ using T1 and T2 sequences were made on all patients to rule out other cranial neuropathies of the cerebellopontine angle (CPA). MRI T2 sequence is the most sensitive in identifying the vessels impinging the facial nerve^8^, ^9^, ^10^, ^11^, ^12^, ^13^, ^14^, ^15^, ^16^, ^17^, ^18^, ^19^, ^20^, ^21^, ^22^, ^23^, ^24^ in the CPA root exit zone (REZ) and more rarely in the entry of the porus. All patients signed a written informed consent.

All procedures were carried out by the same surgical team. Under general anesthesia, the patient is placed in supine position and the head is elevated around 15° by laying it on “rubber donut”. If required (in case of overweight patients with short neck and/or large shoulders), additional elevation of head up to 30° can be obtained by laying additional sheets under the “donut”. The head is turned contralaterally to the surgical side to make the operative site facing upwards and is slightly flexed over the opposite shoulder. The forehead is fastened with adhesive tape; no pin fixation of the skull is used. During the operation, the facial nerve function is assessed by electromyography monitoring^24^ system (NIM 2.0 Medtronic Inc^®^, Minneapolis, MN, USA).

**Figure 1:**
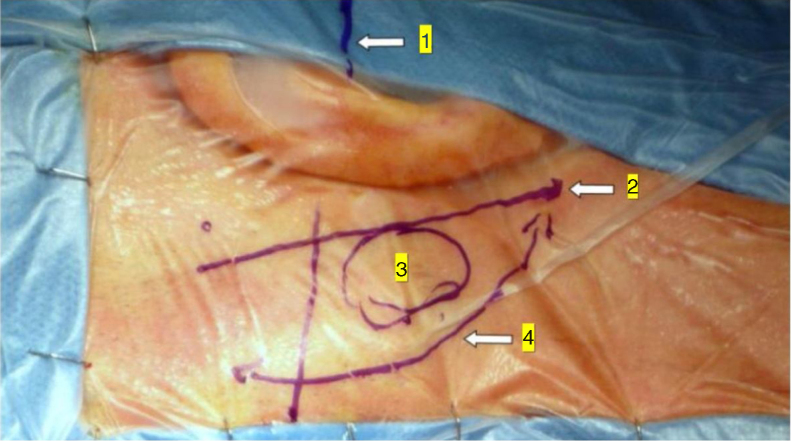
Minimally invasive retrosigmoid approach (right side). The image shows the anatomic landmarks for the surgical access: (1) Frankfurt plane between the external canthus and tragus superior edge; (2) digastrics’ muscle plane; (3) craniotomy site projection; (4) surgical incision.

**Figure 2:**
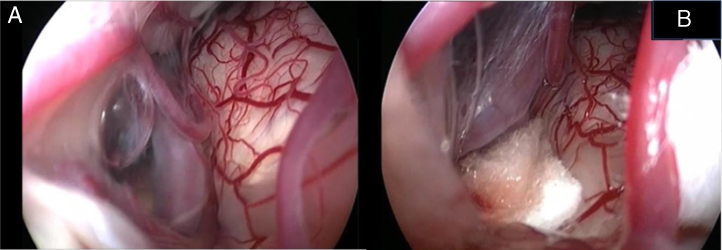
Endoscopic view of the neurovascular conflict before the decompression (A) and after Teflon^®^ sponge interposition between the posterior–inferior cerebellar artery (PICA) and the facial nerve (B).

In the retroauricular area, along the proposed lines of incision (Fig. 1), 5–7 mL of 2% lidocaine with 1:100,000 epinephrine are injected to enhance hemostasis and soft tissue dissection. A 6–8 cm skin incision drawn as an arc is performed with the convex side facing posterior. The incision is placed around 1 cm behind the supposed posterior edge of the craniotomy and 2 fingers to the helix projection to the retro-mastoid region. By placing the incision in such area we can preserve both occipital artery and C2 nerve from trauma. A skin flap is elevated anteriorly. A muscle-periosteal incision is carried out with monopolar cautery. Bone drilling for retrosigmoid craniotomy begins around the emissary vein, by keeping it in the center as a landmark. A large cutting burr is used to start the dissection, while a diamond burr is required in proximity to the dura and the sigmoid sinus to preserve their surfaces. A circular craniotomy from 1.5 to 2 cm of diameter is performed back to the sigmoid sinus. The dura is opened in a V-shape manner, starting just behind the sigmoid sinuses to reduce the necessity of cerebellar mechanical retraction during the access to the IAC. Then, the dural incision is performed 1–2 mm from the craniotomy edges to facilitate dural re-suturing at the end of surgery. Anesthesiology hyperventilation is carried out to reduce CSF intra-cranial pressure and spontaneously retract the cerebellum. Once sufficient cerebellar “relaxation” is achieved, a fine neurosurgical cottonoid or Neuropatch (1.5 cm wide by 5 cm long size) is placed over the cerebellum for protection from possible injuries while introducing instruments. The cisterna magna is opened to gain access to the CPA. With the microscope, the arachnoid surrounding the cranial nerves VIII and lower cranial nerves is dissected. The procedure continues by using the endoscope (Rigid 4 mm, 30°, by KARL STORZ^®^, Tuttlingen, Germany). The acoustic-facial bundle is the central landmark of the CPA and the neurovascular conflict (NVC) area is identified below it at the REZ of the facial nerve. The most common offending vessel is the posterior inferior cerebellar artery (PICA) (Fig. 2A). An additional impinging vessel (anterior inferior cerebellar artery – AICA) is located along the cysternal portion of the facial nerve and sometimes is partially located in the beginning of the internal auditory canal (IAC); a partial drilling of the porus is required and is carried out to allow vessel decompression.

Again, by using the microscope the offending arterial vessel(s) is gently separated from the facial nerve. Sometimes, immediately after facial nerve detachment, a “releasing” trail of electrical stimuli may be noticed on the facial nerve monitoring, which confirms the decompression success.^17^ Teflon^®^ sponges are the interposed between all offending vessel(s) in order to prevent a new neurovascular contact and to insulate the facial nerve (Fig. 2B). If a vein impinges the facial nerve, after vessel detachment, it is carefully coagulated with bipolar forceps. At the end of the decompression procedure, the proper positioning of the teflon is checked with the endoscope. The cottonoids are removed and the CPA filled with saline solution, then the dura is sutured by single re-absorbable stitches to ensure a water proof resistance. Additional connective tissue is placed and fixed by tissuecole. Bone patè, obtained with bone dust (collected during cranial drilling) fixed by fibrin glue, is used to close the craniotomy site. The muscle-periosteal and cutaneous flaps are carefully sutured in layers with single absorbable stitches. A compressive bandage is applied and kept for 4 days. The patient is awoken from general anesthesia, extubated, lead to the postoperative room for a 3 h observation and then returned to the general ward. The patient permanence is 1 week in the hospital. Patients are followed-up periodically for 2 years after surgery.

### Data collection and statistical analysis

Data relating to patients’ age, sex and years from the onset of HFS, HFS side, loop side, vessel involved in the NVC, treatments performed before surgery, co-morbidities, surgical outcomes and complications, and time between surgery and HFS resolution were collected. Data were represented as frequencies and percentages. Statistical analysis was performed using SPSS 10.0 for Windows (SPSS, Chicago, Illinois). Student's *t*-test (*t*), Pearson's and Spearman correlation coefficient (*r*) and Chi-square test (CS) were used when appropriate. A value of *p* < 0.05 was considered as statistically significant.

## Result

Table 1 summarize the main findings of our sample.

**Table 1:** It describes the patient included in the study, the vessel involved in the MVC and results post-surgery.

Patient (sex/age)	Side	Offending vessel (S)	Result
M/60	Left	Vein intracanalicular in pons	Recurrence after 1 year
W/57	Right	PICA + vertebral	Resolution
M/71	Left	AICA	Resolution
W/54	Left	Vertebral	Delayed resolution
W/55	Right	PICA	Resolution
W/47	Left	AICA	Resolution
W/53	Left	PICA	Delayed resolution
M/49	Left	PICA	Resolution
W/59	Left	AICA	Delayed resolution
M/66	Right	Vertebral	Resolution
W/61	Left	PICA	Resolution
M/58	Left	PICA + vertebral	Resolution

**Figure 3:**
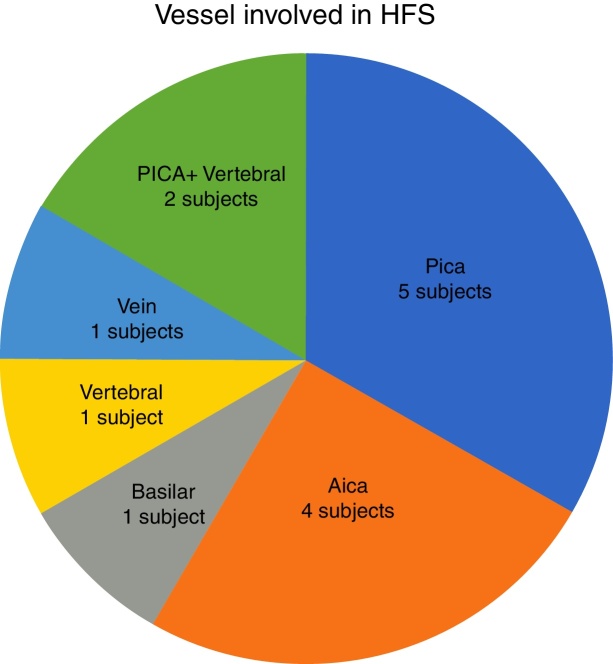
Frequency of the different vessels involved in the neurovascular conflict with the facial nerve in our study group. PICA, posterior–inferior cerebellar artery; AICA, anterior–inferior cerebellar artery.

**Figure 4:**
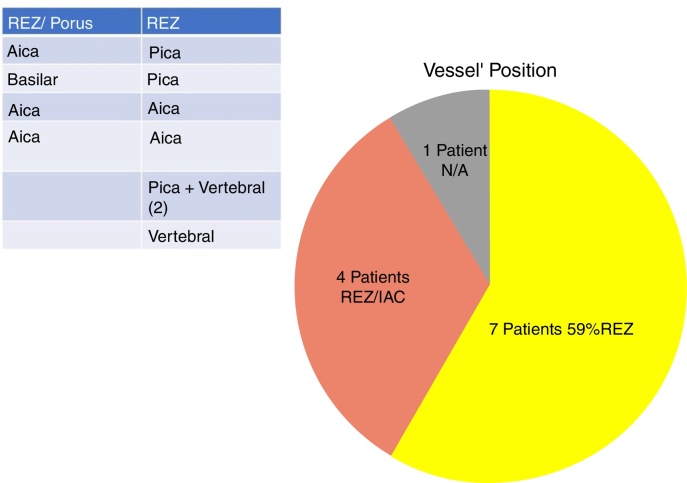
Frequency of the different neurovascular conflict sites in our series. REZ, root entry zone; IAC, internal auditory canal; N/A, non-arterial conflict (vein).

In our study, all patients (100% of cases) presented a correlation between diagnosis and surgical finding.Among our patients, we observed a predominance of left side NVCs (8/12 subjects) with a more frequent involvement (51% vs. 25%) of the posterior–inferior cerebellar artery (PICA) than the anterior–inferior cerebellar artery (AICA). In 2 cases a multiple PICA-vertebral artery impingement on the facial nerve was noticed. The basilar and the vertebral arteries were responsible for the MVC in 1 case each (Fig. 3). An arterial loop impinging the facial nerve was found jutting into the entry of acoustic porous in 33% (4/12 patients) of cases and, involving REZ area only, in 69% (7/12) of subjects (Fig. 4), with significant statistically result (*p* < 0.05). Only one patient affected from HFS with negative MRI for MVC, we identified a vein crossing the facial nerve. The vein was intracanalicular and in contact with pons. Our patients reported a previous clinical history of HFS average presence of 10 years (SD = 7.1; 95% CI 2–29) at the moment of surgery and 66% of them had undergone previous BoTN treatment.

**Figure 5:**
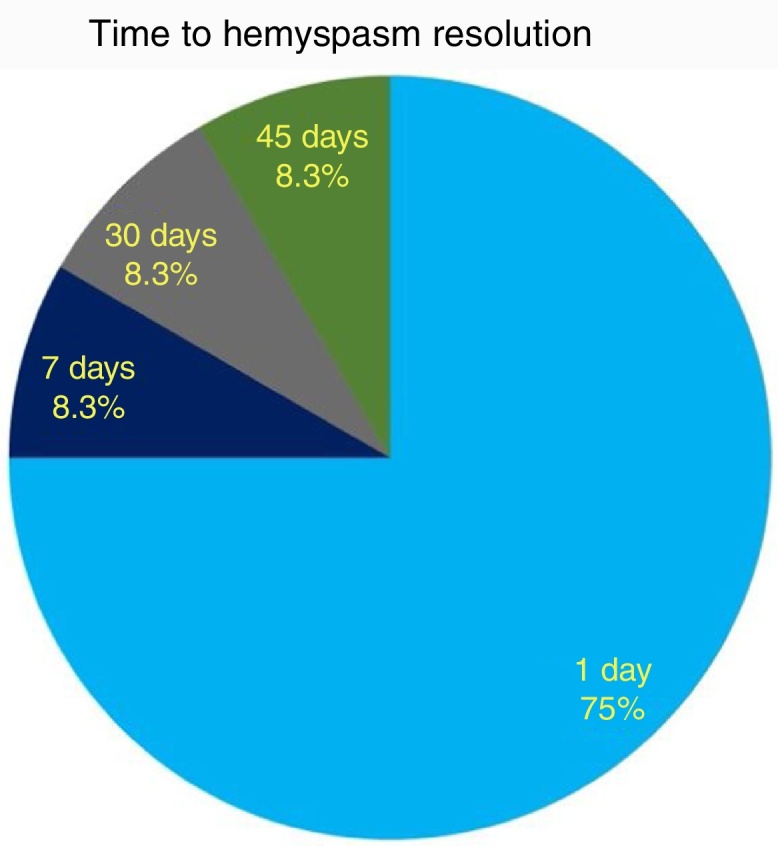
Timing (days after surgery) of hemifacial spasm resolution.

As to functional outcome, we noticed HFS resolution within 24 h after surgery in 9/12 (75%) cases, while all patients resolved their HFS within 45 days (Fig. 5). No correlation between HFS resolution timing and the offending vessel (PICA, AICA or vertebral artery) was found (CS, *p* = 0.7).

The Spearman test identified a significant (*p* < 0.001) negative correlation (Pearson) between preoperative history duration of HFS and postoperative recovery timing, meaning that an increasing of the years of affection correspond to a quick post-surgery recovery.

Postoperative complications were observed in 2/12 (16%) cases: 1 patient has had meningitis and 1 subject a scar infection. The subject that reported meningitis was affected by hypertension; he was treated with i-v antibiotics with meningitis resolution and no long-term sequelae. Among our patients, 25% (3/12) were affected by hypertension and 16% by hyper-triglyceridemia (2/12); none of these conditions influenced HFS recovery timing. No cases of CSF leak, facial palsy or hearing impairment were recorded. During the follow-up, HFS recurrence was noticed 12 months after surgery only in the subject where the impinging vein was identified. No significant correlation was observed between functional outcomes and the specific artery involved in the NVC.

## Discussion

**Figure 6:**
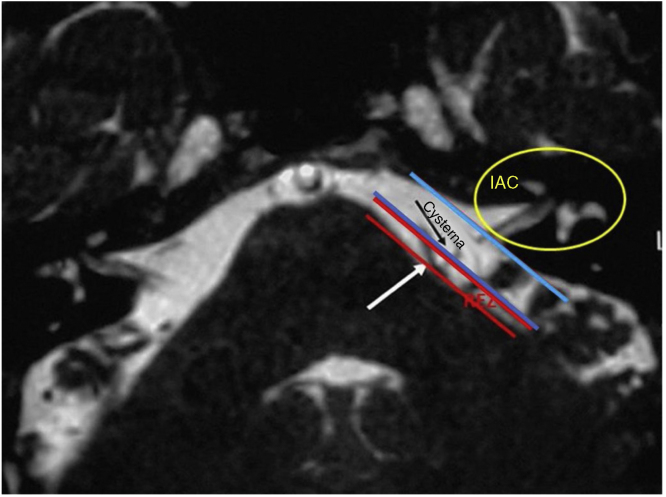
T2 weighted MRI showing the boundaries of the root entry zone (REZ) in the cerebellopontine angle and the internal auditory canal (IAC). Notice the conflict between the vessel (white arrow) and the facial nerve (black arrow) at the REZ.

In our study, a constant association between HFS side and NVC site was noticed, which confirms the concept that a contact between a vessel loop and the facial nerve is responsible for facial spasm (Fig. 6). Nevertheless no significant difference was observed in our sample in the site of nerve compression (REZ vs. IAC), we recorded a higher frequency of porus involvement (33%) with respect to other authors,^2^, ^8^, ^9^, ^10^ The use of combined approach offers a better vision of the course of the offending vessel due to the endoscope view that allows to investigate the vessel position at 360°. The use of a 30° endoscope allowed us a better “around-the-corner” visualization of the IAC in contrast to the microscope's straight linear view.

**Figure 7:**
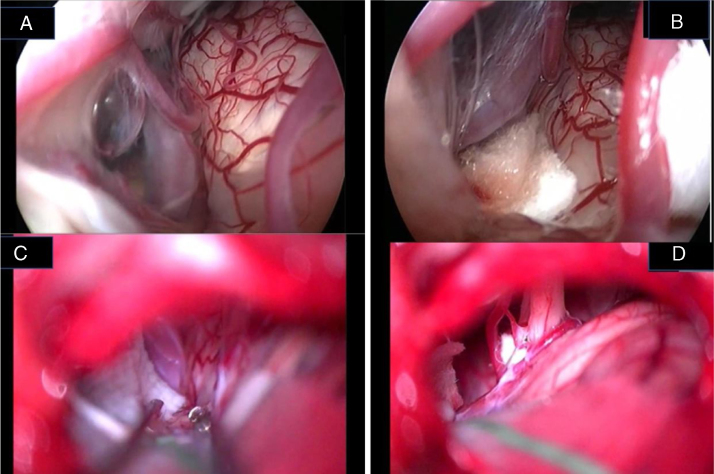
Endoscopic (A, B) and microscopic (C, D) image of a neurovascular conflict before the decompression (A, C) and after Teflon^®^ sheet interposition between the vascular loop and the facial nerve (B, D). It is important to notice the difference in details resolution and anatomical structures definition between endoscopic full-HD technology and microscopic vision. VII, facial nerve; VIII, Statoacoustic nerve; XII, hypoglossal nerve; T, Teflon^®^ sponge; v, vein.

Jannetta in 1977^12^ described the principles of MVD that consists in detaching the offending vascular loop(s) and securing them with a nonabsorbent synthetic sponge with no intentional trauma or disruption of the nerve; after his description a dramatic improvement in surgical outcomes has been reported in the literature. However, this procedure is not without limitations, since several reports have documented failures, recurrences, and complications related to microscopic decompression.^7^, ^8^, ^9^, ^10^, ^11^, ^12^, ^13^, ^14^ Microscope-assisted vascular decompression for HFS is reported to have a mortality rate of 0.2% and an overall complication rate of 5–25% for temporary dysfunction and 2–10% for permanent neurologic impairment.^7^, ^8^, ^9^, ^10^, ^11^, ^12^, ^13^, ^14^ Most complications involve auditory or facial nerve function. The reported rate of auditory nerve impairment is 3–5% for temporary dysfunction and 2–3% for permanent hearing loss.^7^, ^8^, ^9^, ^10^, ^11^, ^12^, ^13^, ^14^, ^23^ Facial nerve impairment occurs temporarily in approximately 4% of patients, whereas 1–2% show permanent facial nerve deficit.^7^, ^8^, ^9^, ^10^, ^11^, ^12^, ^13^, ^14^ The key for a successful decompression surgery in case of HFS includes a precise visualization of all nerve-vessel conflicts and a confirmation of complete nerve decompression at the end of the procedure (Fig. 7A–D). As already reported by Jannetta, the anatomy of the posterior fossa and the limited size of the craniotomy make an adequate visualization of all the facial nerve course and the porus portion difficult by the microscope use only.^12^, ^13^, ^14^

In order to overcome such limitations, some authors proposed the use of the endoscope-microscope combination to identify the real NVC site and reduce complication rates.^15^, ^16^, ^17^, ^18^, ^19^, ^20^, ^21^, ^22^, ^23^ In particular, the senior author of this study already reported the use of the endoscope to assist MVD of the facial nerve and demonstrated an additional 72% accuracy rate in identifying nerve-vessel conflicts without dislocation of the acoustic-facial bundle and cerebellum retraction,^15^, ^16^, ^17^, ^21^, ^22^ which resulted in a decrease in neurological complication rates.

Our experience confirms the advantages of the endoscope-assisted minimally invasive approach in terms of HFS resolution and complication rates with respect to the traditional approach with microscopy only.^7^, ^8^, ^9^, ^10^, ^11^, ^12^, ^13^, ^14^ In particular, 75% of our subjects showed a full HFS resolution 24 h after the surgery, while the remaining patients had a “delayed” recovery. The delayed recovery may be due to the neural distortion and hyperactivity of facial nerve nucleus or due to post-surgery inflammatory phenomena,^24^ even though no definite explanation of this event exists. As to the only case presenting HFS recurrence after surgery, the patient had been treated for a vein impinging on the facial nerve. In that case, bipolar cautery had been used to coagulate the vessel; it is the authors’ opinion that a scar (post-inflammatory event) or re-permeation of the vein was the cause of the HFS recurrence. Our positive results are enabled by the “panoramic” operative view and the “around-the-corner” visualization offered by the endoscope, in addition to the high resolution of modern full-HD technology (Fig. 7A–D). We recommend the 30° angled endoscope as the most appropriate tool for vascular decompression. The minimally invasive approach, opening a small window in the sigmoid area, better protects from cranial pressure fall, by reducing headache and CSF leak risk. Our high rate of success is due not only to the use of the endoscope but also to the minimization of approach and maneuvers. The endoscopic procedure allows the surgeon to precisely identify the structure and to minimize hand movements during the surgery due to the complete vision of surgery area. The safe movements due to the wide understanding of impeachment area diminish the pressure on cerebellum and the risk of central neurological complications. The value of angled vision is of the utmost importance in case of NVC within the IAC in order to perform the decompression maneuvers safely. In case of NVC partially jutting in the IAC, a partial drilling of the canal is required before vascular detachment and the insertion of a small piece of thin polyester urethane sponge (Neuropatch^®^ – B/Braun-Aesculap^®^, Tuttlingen, Germany) between AICA and the facial nerve. Our minimally invasive approach allowed us to perform surgery on even elderly subjects (our oldest patient was 71 years old) without increased postoperative sequel.

## Conclusions

The results of this study confirm the endoscope-assisted minimally invasive retrosigmoid approach is a-safe procedure to visualize the neurovascular conflicts leading to HFS, thus allowing an efficient decompression of the facial nerve with a very low morbidity. Thanks to the reduced post-operative complications this approach should be considered as “gold standard” in patients suffering of hemifacial spasm, regardless of their age.

## Conflicts of interest

The authors declare no conflicts of interest.
